# Linking Individual-Level Facebook Posts With Psychological and Health Data in an Epidemiological Cohort: Feasibility Study

**DOI:** 10.2196/32423

**Published:** 2022-04-07

**Authors:** Peter James, Claudia Trudel-Fitzgerald, Harold H Lee, Hayami K Koga, Laura D Kubzansky, Francine Grodstein

**Affiliations:** 1 Division of Chronic Disease Research Across the Lifecourse Department of Population Medicine Harvard Medical School and Harvard Pilgrim Health Care Institute Boston, MA United States; 2 Department of Environmental Health Harvard TH Chan School of Public Health Boston, MA United States; 3 Department of Social and Behavioral Sciences Harvard TH Chan School of Public Health Boston, MA United States; 4 Lee Kum Sheung Center for Health and Happiness Harvard TH Chan School of Public Health Boston, MA United States; 5 Department of Internal Medicine Rush Medical College Chicago, IL United States; 6 Rush Alzheimer's Disease Center Rush University Chicago, IL United States

**Keywords:** social media, cohort, psychological factors, recruitment, feasibility, middle-aged and older adults, women

## Abstract

**Background:**

Psychological factors (eg, depression) and related biological and behavioral responses are associated with numerous physical health outcomes. Most research in this area relies on self-reported assessments of psychological factors, which are difficult to scale because they may be expensive and time-consuming. Investigators are increasingly interested in using social media as a novel and convenient platform for obtaining information rapidly in large populations.

**Objective:**

We evaluated the feasibility of obtaining Facebook data from a large ongoing cohort study of midlife and older women, which may be used to assess psychological functioning efficiently with low cost.

**Methods:**

This study was conducted with participants in the Nurses’ Health Study II (NHSII), which was initiated in 1989 with biennial follow-ups. Facebook does not share data readily; therefore, we developed procedures to enable women to download and transfer their Facebook data to cohort servers (for linkage with other study data they have provided). Since privacy is a critical concern when collecting individual-level data, we partnered with a third-party software developer, Digi.me, to enable participants to obtain their own Facebook data and to send it securely to our research team. In 2020, we invited a subset of the 18,519 NHSII participants (aged 56-73 years) via email to participate. Women were selected if they reported on the 2017-2018 questionnaire that they regularly posted on Facebook and were still active cohort participants. We included an exit survey for those who chose not to participate in order to gauge the reasons for nonparticipation.

**Results:**

We invited 309 women to participate. Few women signed the consent form (n=52), and only 3 used the Digi.me app to download and transfer their Facebook data. This low participation rate was observed despite modifying our protocol between waves of recruitment, including by (1) excluding active health care workers, who might be less available to participate due to the pandemic, (2) developing a Frequently Asked Questions factsheet to provide more information regarding the protocol, and (3) simplifying the instructions for using the Digi.me app. On our exit survey, the reasons most commonly reported for not participating were concerns regarding data privacy and hesitation sharing personal Facebook posts. The low participation rate suggests that obtaining individual-level Facebook data in a cohort of middle-aged and older women may be challenging.

**Conclusions:**

In this cohort of midlife and older women who were actively participating for over three decades, we were largely unable to obtain permission to access individual-level data from participants’ Facebook accounts. Despite working with a third-party developer to customize an app to implement safeguards for privacy, data privacy remained a key concern in these women. Future studies aiming to leverage individual-level social media data should explore alternate populations or means of sharing social media data.

## Introduction

Substantial research has demonstrated that psychological factors (eg, depression and optimism) and their related biological and behavioral responses are associated with physical health and the risk of chronic diseases of aging [1-3]. The majority of research in this area relies on self-reported assessments of psychological factors, which can be difficult to scale because they can be expensive to administer and time-consuming to complete, and therefore impose substantial burdens on participants and investigators. As a result, investigators are increasingly interested in social media as a novel and convenient platform for obtaining information efficiently in large populations. Developing such low-cost low-burden methods for unobtrusively obtaining assessments of psychological factors at the individual level, which can then be linked with individual health and other types of data, may expand capacity and efficiency for examining how psychological factors impact health.

A small but growing body of research suggests that various psychological factors can be measured using machine learning–derived algorithms that harness social media “big data.” For example, a recent study leveraged text from 5100 public Facebook status updates and built models to assess an individual’s level of psychological well-being (characterized by positive emotions and meaning/purpose in life) embedded within any particular Facebook status update. When comparing algorithm-derived scores with scores annotated by human raters, investigators found moderate correlations of 0.4-0.6 [4]. In another study of 66,732 Facebook users using anonymous data, researchers created an algorithm to estimate other psychological factors similar to Big 5 personality measures; correlations between self-reported and algorithm-based scores derived from social media similarly ranged from 0.4 to 0.5 [5]. Moreover, research has suggested that measures of psychological functioning derived from social media can be used to predict health status. One study examined Twitter posts in 1347 US counties, covering 88% of the US population, and derived measures of psychological functioning using machine learning [6]. Each county was then scored according to levels of negative and positive psychological factors (eg, anger, anxiety, positive emotions, and engagement), and cross-sectional analyses evaluated if these factors were related to county-level rates of heart disease mortality. The psychosocial measures derived from Twitter language were strongly associated with heart disease mortality rates.

However, most studies using social media data to assess psychological functioning in relation to health are ecological (eg, county-level psychological and health data) and cannot link individual-level psychological measures derived from social media to individual-level health outcomes [7,8], a critical methodological element for making causal inferences. Thus, it is important to test the use of social media platforms for individual-level research in cohort studies, where information on demographics and lifestyle, as well as longitudinal data on chronic diseases are available, and enable both the identification of direct relationships and control of potential confounding factors. In particular, Facebook is the most widely used social media platform in the world, with over 2.7 billion users [9]. It has the potential to provide a substantial amount of data on individuals who are posting large quantities of text over extended periods. Moreover, while the majority of Facebook users are young adults, 22% of users are over 45 years of age [10]. To our knowledge, social media approaches to psychological measurement have not been applied in prospective cohort studies of midlife and older adults. Linking psychological factors derived from social media with rich epidemiological data from large prospective studies of midlife and older adults could enable the rigorous and efficient understanding of new perspectives on psychological factors and health outcomes. Therefore, we leveraged the Nurses’ Health Study II (NHSII), an ongoing cohort study of women aged 56 to 73 years in 2020, to examine the feasibility of obtaining participant Facebook data to derive measures of psychological factors.

## Methods

### Study Population

The NHSII is a prospective cohort study that was launched in 1989 among 116,429 US female nurses aged 25 to 42 years at the study onset. At baseline, all participants completed a questionnaire including basic sociodemographic characteristics, lifestyle factors, and medical conditions. The cohort was originally followed using biennial mailed questionnaires to update information on these factors and further assess psychosocial factors. Approximately 60,000 of the women now complete questionnaires online; the follow-up rate since study inception is nearly 90% [11].

The 2017-2018 online questionnaire assessed psychosocial factors, such as optimism, depression, and social support. Items also requested information regarding participants’ use of social media. Specifically, the questionnaire included the following items: “Do you regularly post updates or information on social media (rather than just viewing or *liking* posts)?” Among women who answered “yes,” a follow-up question asked which of the following sites participants used: (1) Facebook, (2) Instagram, (3) Twitter, and (4) other. Ultimately, 18,519 participants reported regularly posting on Facebook. Very few (7%) reported using other social media outlets. Thus, in April 2020, we initiated the study to request Facebook data.

### Protocol

#### Obtaining Facebook Data

When collecting any type of individual-level data, privacy is an important concern, and this concern is potentially magnified when collecting information on individuals that was not originally intended for use in a scientific study. At the time this study was initiated, Facebook was not sharing personal data for investigators to use in the context of scientific research. Thus, such data could only be obtained directly from participants. To reduce privacy concerns, we chose to ask participants only for text from Facebook posts they wrote and did not ask for any other content, including photos, links, or posts written by friends. We worked with third-party software developers at Digi.me to modify a program that would enable participants to obtain their own Facebook data and then to send the text of their posts only to our research team securely. The original Digi.me app enables individuals to obtain and store their own digital content from various sources (eg, finances, health, and social). For our study, we customized the original app, including a process by which Facebook text could be securely transferred from each participant to the NHSII server. We also developed simple instructions for use. The NHSII Digi.me app transferred only the text of participants’ Facebook posts.

#### Ethics Approval

The Brigham and Women’s Hospital Institutional Review Board and Information Security Office conducted an ethical review and a security review of the modified app, as well as the research protocol, and granted approval for the study (2018P002265).

#### Participant Recruitment and Consent

An email invitation was sent to a random subset of the women who reported being regular Facebook users in 2017-2018. The email included a brief description of the study and an informed consent form. If consent was given, participants received an email with instructions for using the NHSII Digi.me app. The app enabled them to (1) securely and privately download their individual Facebook posts, and (2) encrypt and securely send their Facebook text to NHSII servers, to be stored behind a firewall. Participants were informed that Digi.me only enables the secure transfer of information and does not see data at any point.

#### Recruitment Waves

We conducted 3 waves of recruitment. In the initial data collection (Wave 1) occurring in April 2020, we invited a random subset of 40 eligible participants by email. We began the work slowly by inviting a small random subset of eligible participants instead of inviting all participants at once because the Facebook study involved new technology (eg, Digi.me) and potentially large amounts of data transfer. In the second data collection wave (Wave 2), occurring between June and September 2020, we sent the invitation email to a further 269 randomly selected eligible women. In a separate step in September 2020 (Wave 3), we sent an email to participants who had consented to provide their Facebook text but had not transferred their data. The email contained information describing how we had fixed a technological issue, simplified the instructions for the use of Digi.me, and explained that anyone still interested could try to send their Facebook text.

### Measures

Our primary outcomes were the following 2 feasibility measures: the percentage of invited participants who consented to share their Facebook data, and the percentage of invited participants who ultimately provided their Facebook posts. We also conducted an exit survey for eligible women who declined to participate in the Facebook study. These women received a single multiresponse question by email. Women were asked to indicate the reasons why they refused to participate with the following 5 response options: (1) lack of time due to increased work responsibilities, (2) lack of time overall, (3) discomfort using Digi.me because of privacy concerns, (4) discomfort using Digi.me because of dislike of technology/apps, and (5) discomfort about sharing Facebook posts. An open-ended response option was also provided. We chose to do an exit survey to gather data on recruitment and participation in the least burdensome way possible for participants.

### Statistical Analysis

We conducted descriptive analyses (ie, percentages, means, SDs, and frequency tables) examining the demographic characteristics of participants who were eligible for the study, the percentage of women who consented to send Facebook data, the percentage of women who provided Facebook posts, and the responses to the exit survey.

## Results

### Descriptive Data

Among the 18,519 women who reported regular Facebook use (Table 1), ages ranged from 56 to 73 years, with a mean age of 65 years (SD 4.7 years). Overall, 93.8% (17,361/18,519) were White, 73.2% (13,549/18,519) were married, and 82.4% (15,261/18,519) lived in an urban area. The median census home value was US $165,000 (SD US $115,000).

**Table 1:** Characteristics of women eligible for the study to collect Facebook posts (Nurses’ Health Study II, 2020; N=18,519).

Characteristic			Value
Age (years), mean (SD)			65.4 (4.7)
**Race, n (%)**			
	White	17,361 (93.8)	
	African American	160 (0.9)	
	Other	998 (5.4)	
**Marital status, n (%)**			
	Married	13,549 (73.2)	
	Divorced/separated	3,506 (18.9)	
	Widowed	739 (4.0)	
	Missing	725 (3.9)	
Median home value (census tract; US$), mean (SD)			165,000 (115,000)
**Urbanicity of residence, n (%)**			
	Urban	15,261 (82.4)	
	Suburban	1,830 (9.9)	
	Small town/rural	1,424 (7.7)	
	Missing	4 (0.0)	

#### Wave 1 Data Collection

Of the 40 women invited, only 4 (10%) participants signed a consent form. Given the low initial participation rate, we paused recruitment to consider potential reasons and modify our strategy accordingly. We identified several possible concerns regarding the initial lack of participation: (1) the first surge of cases due to the COVID-19 pandemic crisis was occurring at the time, and this may have impacted participation among our nurse participants, and (2) the brief invitation email may not have adequately addressed possible participant concerns regarding the technological burden and personal data sharing involved in the Facebook study. Thus, we modified the study in several ways. First, we excluded women who reported on the 2019 NHSII questionnaire that they were active health care workers. Second, we developed a Frequently Asked Questions factsheet and included it as a link in the invitation emails; this factsheet included more detailed information regarding the steps required to use the technology and the actions we had taken to maximize data security and privacy (eg, encryption). Finally, as described previously, we also included the exit survey inviting women who did not want to participate to provide their primary reasons for not participating.

#### Wave 2 Data Collection

Of the 269 randomly selected participants invited in Wave 2, 48 women (17.8% of Wave 2 invited participants) completed a consent form to participate in sharing their Facebook posts. Among these 48 women, 3 used the Digi.me app to send their Facebook posts (1% of Wave 2 invited participants). Further, 23 women who did not complete the consent form responded to the brief exit survey describing their concerns about participation (Table 2). Each participant could provide more than one response. Of the 23 women, 3 (13.0%) noted that they did not have time to participate, 8 (34.8%) indicated they had concerns regarding privacy, 1 (4.3%) indicated not liking the use of apps, and 12 (52.2%) indicated they did not want to share all the information in their Facebook posts. In addition, 6 women (26.1%) provided written comments in the space for “other concerns;” these mostly involved comments that they had stopped using Facebook or used it only in a very limited way. Further, on receiving the Facebook text from 3 participants, we identified some problems in the data transfer; the 3 women also emailed that they found the directions for using Digi.me somewhat complex. Thus, before initiating a third wave of invitations, we fixed the data transfer issue and also simplified the instructions for using Digi.me.

**Table 2:** Reasons for choosing not to participate in the study to send Facebook text (Nurses’ Health Study II; N=23).

Reason provided in the survey^a^	Value, n
I am working more than usual and do not have time	2
I would have been interested in participating if I had more time	1
I don’t feel comfortable using digi.me because of privacy concerns	8
I don’t feel comfortable using digi.me because I do not like technology/apps	1
I don’t feel comfortable sharing my Facebook posts	12
Other	6

^a^Women were requested to mark all responses that were relevant to them.

#### Wave 3 Data Collection

In the third data collection that occurred in September 2020, we sent an email to a total of 49 women (15.9% of all invited participants) who had consented to provide their Facebook text but had not transferred the data. The email explained that we had fixed a technological issue and simplified the instructions for use of Digi.me, and that anyone still interested could send their Facebook text. However, we received no additional data transfers.

Of the 309 participants invited overall, 52 consented (16.8%) and 3 attempted to transfer data (1.0%). On carefully considering the low rate of participation, we decided to end the Facebook study and did not send invitations to the remaining eligible women.

## Discussion

The goal of this study was to examine the feasibility of using social media data to assess psychological factors, and ultimately examine if these passively measured factors were associated with health outcomes. We queried middle-aged to older women in an ongoing cohort study, the NHSII, on their use of social media. A substantial number reported regularly posting on Facebook (approximately 28%), and few reported using other platforms (eg, 5% Instagram and 2% Twitter) [12]. Working with an industry partner, we developed a customized app to enable participants to download their Facebook data and to transfer Facebook text to the cohort servers using highly secure processes. However, despite providing information about their health and behavior for over three decades on biennial questionnaires and giving biospecimens (eg, blood and toenails) on more than one occasion, very few women agreed to share their Facebook data for cohort research. On exit surveys, women noted that the key issues were concerns about sharing social media data and worries about privacy.

Much of the research to date considering social media data in relation to health has relied on ecological-level data, namely using county-level aggregated social media data from Twitter and linking the data to measures of health status from the same counties [6,13]. Other work has used a computational approach to identify publicly available social media data from the profiles of users who self-disclose health status information in some way, without any means to verify the health information [14,15] and with little available information on other potential confounding factors (eg, sociodemographics, health status, and lifestyle factors). Such work can provide important insights and novel strategies for identifying public health concerns (eg, rising rates of depression) [16]. However, additional insights may be gained by linking social media–derived measures of psychological or behavioral functioning with individual-level health outcomes.

The few early studies seeking to collect this type of data seemed encouraging. For example, in a study of patients in an emergency department, researchers approached individuals over a 26-month period to invite participants to share Facebook postings as well as data from medical records [17]. Of 11,224 individuals who were approached, 2903 consented and were eligible. Among these, 1175 participants (44%) were able to log into their Facebook accounts and share their data with the investigators through an app. Notably, the mean age of consenting individuals was 29 years, and the majority were Black women. Another study recruited 223 participants, primarily psychiatric patients, to participate in a study examining if Facebook data could differentiate participants with different psychiatric diagnoses, drawing on individual-level psychiatric data from medical records [14]. The mean age of the participants was 24 years, with majority being female and White individuals. In a similar study, other authors recruited participants from an emergency room to obtain social media data and access to their electronic health records. Of the 5256 individuals approached, 2717 (52%) were Facebook and/or Twitter users, and among the social media users, 1432 (53%) agreed to participate in the study. Of these participants, 1008 (71%) consented to share their social media data for the purpose of comparing the data with their electronic medical records [7]. Participants who were willing to share their social media data were younger (29.1 years among sharers vs 31.9 years among nonsharers), more likely to be Black, less likely to be White or Asian, and more likely to frequently post on social media. Clearly, there are many differences between these studies and our study, from the average age of participants to the very short-term requirements and data storage of these studies (ie, in contrast to NHSII research in which data are continually stored and utilized for decades).

In a recent study seeking to characterize the willingness of individuals to share 19 different types of digital data (eg, email, fitness tracker, voting history, and Google search) [8], of 595 individuals at an academic urban emergency department who were invited to participate, 206 consented and about half of these expressed willingness to share some form of digital data. The majority of participants were young (70% were less than 44 years old), female, and Black. However, it is worth noting that among those who did participate, fewer than 50% of participants reported current willingness to share Facebook (or similar) digital data, and many identified substantial concerns around potential data and privacy breaches related to sharing digital data in general. As noted in this study, concerns about privacy may have been exacerbated after 2018, when the public learned that some companies were able to access the data of many millions of individuals’ Facebook accounts without their permission.

In this study, many of the women who did consent to provide Facebook data subsequently did not download and send their data to the cohort. In the typical NHSII protocol, women provide data by filling out a detailed questionnaire every other year, which can be sent via mail or completed online. In the substudy, women needed to engage in multiple steps to provide their data, including downloading an app, creating an account on a cloud provider, linking this account to the app, and downloading and then transferring their data to the NHSII servers. Thus, the process for participating in this study required comfort with digital interfaces more than most prior data collection activities in the cohort.

In addition, women identified privacy concerns as a barrier to participation. Prior to conducting this research, we were highly sensitive to potential concerns women in our cohort might have about data privacy, particularly in the aftermath of reporting on breaches of data privacy in the context of Facebook in 2018. To reduce concerns about breaches, we worked with Digi.me, a company dedicated to facilitating individuals’ control and use of their own digital data with safeguards for privacy. The app made it possible for women to download their Facebook data and then to securely transfer the relevant data to our research database. As noted above, to reduce concerns about privacy, we committed to obtaining only text, rather than images or posts from friends. Together with Digi.me, we invested substantial time and effort to customize the app to make it possible to curate the data we obtained, as well as to provide simple instructions and maximize data privacy and security. Despite these efforts, the participation rate in our study was low. Thus, it is possible that social media research may be better suited to populations who more frequently use digital apps, which may explain the higher participation rates in previous studies of younger populations [7,14,17].

The limitations of this study include the potential lack of generalizability of our findings to other populations. Our study population was made up of women who were 56 to 73 years of age, primarily white, and educated professionals. Therefore, care should be taken in extrapolating our results to other demographic groups. In addition, the potential participants were members of a long-term cohort, who have provided a large amount of personal and health data to the study previously, which may have influenced their willingness to contribute Facebook data. However, as the participants, who have developed a relationship of trust with the research for decades, did not feel comfortable sharing their Facebook data, the results would plausibly be worse in newer cohorts. Our exit survey was brief and therefore somewhat limited in that we could not tease out the exact or specific reasons why participants did not feel comfortable sharing their Facebook posts. Eventually, in this type of research, one limitation of deriving psychological factors from Facebook data is that Facebook, or other organizations, may commoditize the data for uses that are not directed toward benefitting society, for instance, targeted advertising. Although our study protocol was not successful in obtaining Facebook data or developing algorithms for deriving psychological factors using Facebook data, other researchers should be aware of the potential ethical implications of building these tools and using these data for research. Finally, another limitation of our approach is that recruitment took place during the COVID-19 pandemic. That said, the study protocol was entirely web-based, and we excluded nurses who were active health care workers, so it is unclear how the pandemic might have affected participation.

The question remains as to the range of paths for scientific research to leverage individual-level social media data to inform our understanding of health and well-being among midlife and older individuals. Future work seeking to leverage social media data to understand health will need to carefully consider the populations under study, especially barriers in recruiting older individuals who may be less familiar with such technology, and solutions for enhancing participation. In addition, researchers should conduct qualitative work to understand better how participants interact with social media, including what type of information they are willing to disclose and how they might curate their image on social media. Besides the challenges we identified, social media platforms and apps are changing rapidly, and the frequency of use and the tools developed for accessing platforms may evolve quickly. This is an area of research that must be fast-paced by definition, while infrastructure (especially processes for ensuring ethical research practices) for conducting medical research necessarily moves much more slowly. In conclusion, individual-level research using social media data will best proceed with a clear understanding of the barriers and challenges existing in specific populations and in doing research in a rapidly changing data environment.

## Abbreviations

NHSII: Nurses’ Health Study II
